# Ultrasonographic Measurement of the Renal Cortical Thickness‐to‐Abdominal Aortic Ratio in Healthy Rabbits

**DOI:** 10.1002/vms3.70455

**Published:** 2025-07-18

**Authors:** Zahra Sadat Yousefsani, Ali Mirshahi, Mohammad Azizzadeh, Amir Nadimi Khorasani

**Affiliations:** ^1^ Private Veterinarian Mashhad Iran; ^2^ Faculty of Veterinary Medicine Department of Clinical Sciences Ferdowsi University of Mashhad Mashhad Iran

**Keywords:** aorta, kidney, rabbits, renal cortical thickness, ultrasonography

## Abstract

**Background:**

Ultrasonography is a preferred diagnostic tool for renal disease in clinical practice. Kidney size is a commonly used morphological parameter in renal assessment; however, it lacks sensitivity for early disease detection. Cortical parameters, particularly cortical thickness, correlate more strongly with early renal disease, as the cortex plays a key role in filtration and is highly vulnerable to injury. Rabbits, widely kept as pets, have distinct renal anatomy and are prone to kidney diseases, making them a relevant model for renal studies. Previous studies have shown that ultrasonography can effectively measure kidney size in rabbits, correlating with body weight.

**Objectives:**

This study aimed to establish a normative ratio of renal cortical thickness to the internal diameter of the abdominal aorta in healthy rabbits.

**Methods:**

Sixty healthy adult male mixed‐breed rabbits underwent ultrasonographic evaluation. Renal cortical thickness and abdominal aortic diameter were measured, ensuring that only rabbits without clinical or ultrasonographic signs of kidney disease were included.

**Results:**

Pearson correlation analysis revealed a positive correlation between renal cortical thickness, aortic diameter and body weight. However, the ratio of renal cortical thickness to aortic diameter remained independent of body weight.

**Conclusions:**

This study established a normative reference range for renal cortical thickness relative to aortic diameter in rabbits, providing a novel diagnostic parameter for renal assessment. The confidence intervals for the ratio were 0.82 (0.77–0.87) to 1.35 (1.30–1.40) for the left kidney and 0.79 (0.74–0.84) to 1.33 (1.27–1.38) for the right kidney, marking the first report of this method in the literature.

## Graphical abstract

This study establishes the renal cortical thickness‐to‐aortic diameter ratio as a novel, body weight‐independent ultrasonographic index in healthy rabbits. The findings provide a normative reference range, offering a reliable, non‐invasive tool for renal assessment. This is the first study to report this method in rabbits.

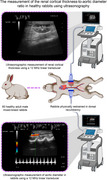

## 1 Introduction

Although kidney size is a widely used morphological parameter, it lacks sufficient sensitivity for the early detection of kidney diseases. In contrast, cortical parameters exhibit a stronger correlation with renal disease in the context of early diagnosis (Mounier‐Vehier et al. 2002). The kidney is highly susceptible to hypoxia, utilising only up to 10% of the oxygen supplied by the renal artery. While the renal cortex receives the majority of the blood supply, the renal medulla receives only 10% to 15% (Wang et al. 2022). Moreover, due to arteriovenous oxygen shunting and the absence of key enzymes required for anaerobic glycolysis, the renal cortex is more vulnerable to injury than the renal medulla (Epstein 1997; Levy and Imperial 1961). A reduction in cortical thickness is commonly observed in various kidney diseases, including chronic kidney disease (CKD) and is associated with decreased renal function. Ultrasonographic measurement of cortical thickness has been directly linked to renal function in CKD patients (Beland et al. 2010). Rabbits are a valuable species widely kept as pets (Miller et al. 2014). Rabbits have typical bean‐shaped kidneys located in the sub‐lumbar retroperitoneal space. Like most mammals, the right kidney is positioned more cranially than the left (Fisher 2006). Unlike the kidneys of many other mammals, the kidneys of rabbits have a single papilla and one calyx forming the renal pelvis that enters the ureter on each side (Fisher 2006; Meredith and Lord 2014). Various kidney diseases affect rabbits, including renal insufficiencies, urinary stones and renal calcification (Varga 2014). Ultrasonography is a crucial diagnostic imaging technique in both clinical and research settings, offering a non‐invasive, real‐time and cost‐effective method for assessing kidney health. Unlike advanced imaging modalities such as computed tomography (CT) or magnetic resonance imaging (MRI), ultrasonography does not require general anaesthesia, minimising stress on animals and aligning with the 3Rs principles (Replacement, Reduction, Refinement) in animal research. It provides detailed visualisation of renal size, shape and internal structures, facilitating the diagnosis of renal disease while reducing invasiveness (Jaturanratsamee et al. 2023; Debruyn et al. 2012; Balls 2009).

In rabbits, ultrasonography has been shown to reliably measure kidney size, with both left and right kidney dimensions positively correlating with body weight (Banzato et al. 2015). In dogs, a previous study aimed to establish normal ultrasonographic reference ranges for renal cortical thickness, independent of age‐related changes in body size. The findings revealed that renal cortical thickness values were associated with body weight, while the renal cortical thickness to aortic diameter ratio showed no significant variation across different body weight groups (Lee et al. 2022). Additionally, this ratio has been used as a non‐invasive quantitative tool to assess kidney pathology in dogs with acute or CKD (Choo et al. 2023).

While the renal cortical thickness to aortic diameter ratio has been studied in other species, including dogs, it has not been previously explored in rabbits. In other animals, this ratio has proven to be a reliable index for evaluating renal cortical thickness independent of body weight. The primary objective of this study was to determine the ratio of ultrasonographic measurements of renal cortical thickness to the internal diameter of the abdominal aorta. This ratio was assessed across different body weights in healthy rabbits to establish a reliable index for evaluating renal cortical thickness, regardless of weight.

## 2 Materials and Methods

**FIGURE 1:**
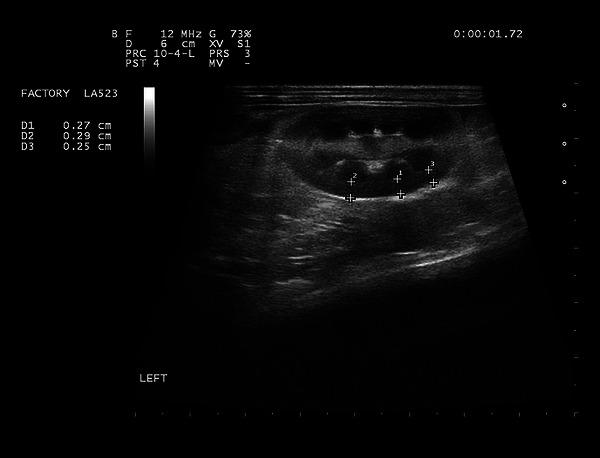
Sagittal ultrasonographic image of the left kidney. At three points where the broad base of the medullary pyramid was clearly apparent, the renal cortical thickness was measured as the shortest distance between the leading edge of the base and the trailing edge of the renal capsule.

**FIGURE 2:**
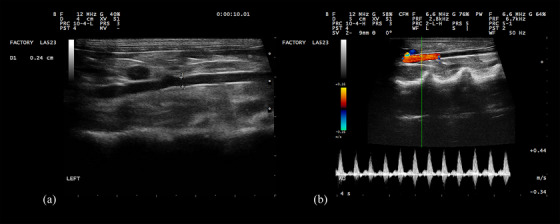
(a) Sagittal ultrasonographic image of an abdominal aorta in rabbit, between electronic cursors is the internal diameter of the abdominal aorta caudal to the left renal artery. (b) Colour and spectral doppler ultrasonogram of abdominal aorta in rabbit.

This study was approved by the Faculty of Veterinary Medicine Ethics Committee of the Ferdowsi University in Mashhad, Iran (IR.UM.REC.1401.268). The study was conducted on 60 healthy adult male mixed‐breed rabbits, weighing from 1.1 to 2.4 kg (mean ± SD, 1.63 ± 0.26 kg) with no history of disease. The rabbits were acquired from a licensed pet store and housed under standardised husbandry conditions to ensure consistency. Inclusion criteria included the absence of urinary‐related clinical symptoms, normal abdominal ultrasonographic findings and unremarkable physical examination and laboratory blood test results. Rabbits were excluded if abnormalities were detected in the kidneys or other abdominal organs. Ultrasound examination of both kidneys was performed to evaluate normal renal cortical echogenicity (hypoechoic or isoechoic to the liver), normal shape and margin, normal medullary echogenicity (hypoechoic to the renal cortex), a clearly delineated corticomedullary junction and the absence of hyperechoic or hypoechoic focal lesions. The abdominal aorta was examined ultrasonographically to confirm the absence of abnormalities such as aortic wall irregularities, thrombosis or calcification. Rabbits with any of these abnormalities were excluded from the study. The rabbits included in this study showed no symptoms of kidney disease, no abnormalities on abdominal ultrasound—particularly of the kidneys—no serum abnormalities (creatinine and urea) and no abnormal complete blood count findings (neutrophilia, eosinopenia, lymphopenia, thrombocytosis or monocytosis). No notable anamnesis, including recent medication use or systemic signs such as obesity, reduced appetite, weight loss, alopecia or abnormal coat condition, was observed. Images were taken with the animals physically restrained in dorsal recumbency. The rabbits were prepared by carefully clipping the abdominal hair, followed by the application of ultrasound gel. Kidneys were imaged in both sagittal and transverse planes, but measurements were taken from the sagittal plane. Renal cortical thickness was measured in the sagittal plane at three points where the broad base of the medullary pyramid was clearly visible. The measurement was taken as the shortest distance between the leading edge of the medullary pyramid base and the trailing edge of the renal capsule (Figure 1). The intraluminal diameter of the abdominal aorta was measured in the midsagittal plane, caudal to the branch of the left renal artery. A still image was reviewed from cine loop frames to determine the maximum intraluminal diameter for accurate measurement (Figure 2).

These measurements were averaged, and the mean value for each kidney was used to calculate individual values.

Ultrasound examinations were performed using an Esaote Mylab 30 gold CV system equipped with a 12 MHz linear transducer. A standard imaging protocol was followed, and each examination took approximately 4–6 min per animal. To reduce interobserver variability, all examinations and ultrasonography were performed by the same operator.

### 2.1 Statistical Analysis

The Shapiro–Wilk test was used to assess the distribution of renal cortical thickness in both the left and right kidneys, as well as the intraluminal diameter of the abdominal aorta. The relationship between body weight and aortic diameter with renal cortical thickness measurements in the left and right kidneys was evaluated using the Pearson correlation test. Additionally, the Pearson correlation test was applied to evaluate the relationship between body weight and the ratio of renal cortical thickness in the left and right kidneys to the aortic diameter.

All statistical analyses were conducted using SPSS software, version 21. The reference interval for the left and right renal cortical thickness to aortic diameter ratio was calculated parametrically using MedCalc software, version 13. A *p* value of < 0.05 was considered statistically significant.

## 3 Results

In the present study, 60 adult rabbits underwent ultrasound and imaging was done from the kidneys on both sides as well as from the abdominal aorta in the longitudinal view. Table 1 provides descriptive statistics based on the obtained measurements. The Pearson correlation test was utilised to determine the correlation between body weight, renal cortical thickness and aortic diameter. The renal cortical thickness and the aorta diameter showed a positive correlation with body weight. This means that with increasing weight, the renal cortical thickness and the aortic diameter increase (Table 2). The correlation between renal cortical thickness and aorta diameter was investigated using the Pearson correlation test. The renal cortical thickness showed a positive correlation with aorta diameter. This means that with increasing aorta diameter, the renal cortical thickness increases (Table 2). The correlation between body weight and the ratio of the left and right renal cortical thickness to aorta diameter was evaluated by the Pearson correlation test. The ratio of left and right renal cortical thickness to aorta diameter did not show a significant correlation with weight. This suggests that the ratio of the left and right renal cortical thickness to aorta diameter has been able to create an index without dependence on the body weight of the animal. Descriptive statistics of the ratio of the left and right renal cortical thickness to the aorta are provided in Table 3. The normal range of this ratio was expressed with a 90% confidence interval (CI) between 0.82 (0.77–0.87) and 1.35 (1.30–1.40) for left kidney and between 0.79 (0.74–0.84) and 1.33 (1.27–1.38) for right kidney in healthy rabbits.

**TABLE 1:** Ultrasonographic measurements of the renal cortical thickness and abdominal aorta in mixed‐breed rabbits (*N* = 60).

	Mean	Standard deviation	Minimum	Maximum
**Left kidney**				
Renal cortical thickness (cm)	0.27	0.03	0.21	0.32
**Right kidney**				
Renal cortical thickness (cm)	0.26	0.03	0.21	0.33
Aorta (cm)	0.25	0.03	0.20	0.32
**Weight (kg)**	1.63	0.26	1.05	2.24

**TABLE 2:** Correlation between weight and renal cortical thickness and aortic diameter.

	Weight (kg)		Ao (cm)	
	Correlation coefficient (*r*)	*p* value	Correlation coefficient (*r*)	*p* value
Left renal cortical thickness (cm)	0.32	0.013	0.275	0.033
Right renal cortical thickness (cm)	0.58	< 0.001	0.316	0.014
Ao (cm)	0.441	< 0.001		

**TABLE 3:** Descriptive statistics of the ratio of the left and right renal cortical thickness to aorta.

	Mean	Standard deviation	Minimum	Maximum	Upper limit (90% CI)	Lower limit (90% CI)
**Left kidney**						
Renal cortical thickness/aorta	1.08	0.14	0.79	1.50	0.82 (0.77–0.87)	1.35 (1.30–1.40)
**Right kidney**						
Renal cortical thickness/aorta	1.06	0.14	0.81	1.37	0.79 (0.74–0.84)	1.33 (1.27–1.38)

## 4 Discussion

In the present study, renal cortical thickness and the intraluminal diameter of the abdominal aorta, caudal to the branch of the left renal artery, were measured to determine their relationship with body weight. The results indicated that as rabbits gain weight, both the renal cortical thickness and the intraluminal diameter of the abdominal aorta increase. Furthermore, a correlation was found between renal cortical thickness and aortic diameter, showing that an increase in cortical thickness corresponds with an increase in aortic diameter. The ratio of renal cortical thickness to aortic diameter was also calculated, suggesting its potential use as an index for evaluating renal cortical thickness in rabbits of varying weights. This ratio provides a reliable, non‐invasive tool for monitoring renal health, independent of body weight. According to our review of the existing literature, this ratio method has not been documented previously in rabbits.

In this study, we evaluated the relationship between renal cortical thickness and body weight, finding a positive correlation for both kidneys. This result is consistent with previous studies, which have also demonstrated a positive correlation between body weight and kidney size in rabbits (Banzato et al. 2015). Furthermore, we observed a positive correlation between body weight and the abdominal aortic diameter just caudal to the left renal artery. This finding aligns with previous research conducted on rabbits (Ghaffari et al. 2023) and dogs (Lee et al. 2022; Ghavidel et al. 2019).

In another study, 60 dogs were evaluated for renal cortical thickness using ultrasound. The dogs were separated into two groups based on their body condition score. Significant positive correlations were observed between renal cortical thickness and both body weight and body surface area, with stronger correlations noted in dogs with normal body condition scores. Multiple regression analysis revealed that renal cortical thickness increased with body weight and body surface area but decreased with body condition score. It also supported that the renal cortical thickness to abdominal aortic diameter ratio was independent of body weight (Lee et al. 2022).

Another study assessed whether differences in renal cortical thickness, considering body weight and body condition score, could differentiate normal dogs from those with acute or chronic renal disease. Results showed that renal cortical thickness values fell outside normal ranges in dogs with CKD or acute kidney injury. Specifically, the renal cortical thickness to aorta ratio was found to be smaller in CKD patients and larger in acute kidney injury patients compared to normal dogs. Similar to our research, this study highlights the critical role of renal cortical thickness as a determinant of kidney health (Choo et al. 2023).

One of the limitations of the current study was its inaccessibility to exclude rabbits with kidney disorders. Thus, we could not present a cut‐off for the pathologic changes in the renal cortical thickness.

## 5 Conclusion

The ratio derived from this study can serve as a normative value for assessing renal cortical thickness relative to aortic diameter in rabbits. The normal range of this ratio was expressed with a 90% CI between 0.82 (0.77–0.87) and 1.35 (1.30–1.40) for left kidney and between 0.79 (0.74–0.84) and 1.33 (1.27–1.38) for right kidney, in 60 healthy rabbits. According to our review of the existing literature, this ratio method has not been documented previously in rabbits.

## Author Contributions

**Zahra Sadat Yousefsani**: conceptualization, software, data curation, investigation, validation, visualization, writing – original draft. **Ali Mirshahi**: conceptualization, data curation, investigation, validation, visualization, writing – original draft, methodology, supervision, funding acquisition, project administration, resources, writing – review and editing. **Mohammad Azizzadeh**: formal analysis, software, investigation, validation, visualization, writing – review and editing, methodology, conceptualization. **Amir Nadimi Khorasani**: conceptualization, software, data curation.

## Ethics Statement

This study was approved by the Faculty of Veterinary Medicine ethics committee of the Ferdowsi University in Mashhad, Iran (IR.UM.REC.1401.268).

## Conflicts of Interest

The authors declare no conflicts of interest.

## Peer Review

The peer review history for this article is available at https://publons.com/publon/10.1002/vms3.70455.

## Data Availability

The data that support the findings of this study are available from the corresponding author upon reasonable request.

## References

[vms370455-bib-0001] Balls, M. 2009. The Three Rs and the Humanity Criterion: Reduction, Refinement, Replacement. FRAME.

[vms370455-bib-0002] Banzato, T. , L. Bellini , B. Contiero , P. Selleri , and A. Zotti . 2015. “Abdominal Ultrasound Features and Reference Values in 21 Healthy Rabbits.” Veterinary Record 176, no. 4: 101. 25362002 10.1136/vr.102657

[vms370455-bib-0003] Beland, M. D. , N. L. Walle , J. T. Machan , and J. J. Cronan . 2010. “Renal Cortical Thickness Measured at Ultrasound: Is It Better Than Renal Length as an Indicator of Renal Function in Chronic Kidney Disease?” American Journal of Roentgenology 195, no. 2: W146–W149. 20651174 10.2214/AJR.09.4104

[vms370455-bib-0004] Choo, D. , S. S. Kim , D. Kwon , K. Lee , and H. Yoon . 2023. “Ultrasonographic Quantitative Evaluation of Acute and Chronic Renal Disease Using the Renal Cortical Thickness to Aorta Ratio in Dogs.” Veterinary Radiology & Ultrasound 64, no. 1: 140–148. 36049077 10.1111/vru.13154

[vms370455-bib-0005] Debruyn, K. , H. Haers , A. Combes , et al. 2012. “Ultrasonography of the Feline Kidney: Technique, Anatomy and Changes Associated With Disease.” Journal of Feline Medicine and Surgery 14, no. 11: 794–803. 23087005 10.1177/1098612X12464461 PMC11112170

[vms370455-bib-0006] Epstein, F. H. 1997. “Oxygen and Renal Metabolism.” Kidney International 51, no. 2: 381–385. 9027710 10.1038/ki.1997.50

[vms370455-bib-0007] Fisher, P. G. 2006. “Exotic Mammal Renal Disease: Diagnosis and Treatment.” Veterinary Clinics: Exotic Animal Practice 9, no. 1: 69–96. 16407080 10.1016/j.cvex.2005.10.002

[vms370455-bib-0008] Ghaffari, T. , A. Mirshahi , A. A. Sarchahi , and M. Azizzadeh . 2023. “Ultrasonographic Measurement of the Adrenal Gland‐to‐Abdominal Aortic Ratio as a Valuable Method of Estimating Normal Adrenal Size in Rabbits.” Anatomia, Histologia, Embryologia 52, no. 2: 234–240. 36259619 10.1111/ahe.12875

[vms370455-bib-0009] Ghavidel, M. , A. Mirshahi , M. Azizzadeh , and J. Khoshnegah . 2019. “Evaluating the Correlation Between Adrenal Gland Dimensions and Aortic Diameter in Healthy Dogs.” Anatomia, Histologia, Embryologia 48, no. 4: 325–333. 31006908 10.1111/ahe.12443

[vms370455-bib-0010] Jaturanratsamee, K. , N. Choisunirachon , K. Soontornvipart , D. Darawiroj , N. Srisowanna , and C. Thanaboonnipat . 2023. “Ultrasonographic Kidney Length‐to‐Abdominal Aortic Diameter for the Diagnosis of Feline Chronic Kidney Disease: A Preliminary Study.” Veterinary World 16, no. 5: 1114–1121. 37576749 10.14202/vetworld.2023.1114-1121 PMC10420722

[vms370455-bib-0011] Lee, J. , S. S. Kim , D. Kwon , Y. Cho , K. Lee , and H. Yoon . 2022. “Measurement of Renal Cortical Thickness Using Ultrasound in Normal Dogs: A Reference Range Study Considering Bodyweight and Body Condition Score.” Veterinary Radiology & Ultrasound 63, no. 3: 337–344. 35023240 10.1111/vru.13053

[vms370455-bib-0012] Levy, M. N. , and E. S. Imperial . 1961. “Oxygen Shunting in Renal Cortical and Medullary Capillaries.” American Journal of Physiology‐Legacy Content 200, no. 1: 159–162. 10.1152/ajplegacy.1961.200.1.159 13761637

[vms370455-bib-0013] Meredith, A. , and B. Lord . 2014. BSAVA Manual of Rabbit Medicine. British Small Animal Veterinary Association.

[vms370455-bib-0014] Miller, I. , C. Rogel‐Gaillard , D. Spina , L. Fontanesi , and A. M. de Almeida . 2014. “The Rabbit as an Experimental and Production Animal: From Genomics to Proteomics.” Current Protein & Peptide Science 15, no. 2: 134–145. 24555894 10.2174/1389203715666140221115135

[vms370455-bib-0015] Mounier‐Vehier, C. , C. Lions , P. Devos , et al. 2002. “Cortical Thickness: An Early Morphological Marker of Atherosclerotic Renal Disease.” Kidney International 61, no. 2: 591–598. 11849401 10.1046/j.1523-1755.2002.00167.x

[vms370455-bib-0016] Varga, M. 2014. Textbook of Rabbit Medicine. Butterworth‐Heinemann.

[vms370455-bib-0017] Wang, B. , Z. L. Li , Y. L. Zhang , Y. Wen , Y. M. Gao , and B. C. Liu . 2022. “Hypoxia and Chronic Kidney Disease.” EBioMedicine 77: 103942. 35290825 10.1016/j.ebiom.2022.103942 PMC8921539

